# Molecular Properties of Phosphodiesterase 4 and Its Inhibition by Roflumilast and Cilomilast

**DOI:** 10.3390/molecules30030692

**Published:** 2025-02-04

**Authors:** Hyun Jeong Kwak, Ki Hyun Nam

**Affiliations:** 1 Department of Bio and Fermentation Convergence Technology, Kookmin University, Seoul 02707, Republic of Korea; 2 College of General Education, Kookmin University, Seoul 02707, Republic of Korea

**Keywords:** PDE4, phosphodiesterase 4, inhibitor, roflumilast, cilomilast, crystal structure

## Abstract

Phosphodiesterase 4 (PDE4) catalyzes cyclic adenosine monophosphate (cAMP) hydrolysis, playing a crucial role in the cAMP signaling pathway. cAMP is a secondary messenger involved in numerous physiological functions, such as inflammatory responses, immune responses, neural activity, learning, and memory. PDE4 inhibition is important for controlling anti-inflammatory and neuroprotective effects. In this review, we provide a comprehensive overview of the molecular functions and properties of human PDE4s. The study presents detailed sequence information for the PDE4 isoforms and the structural properties of the catalytic domain in members of the PDE4 family. We also review the inhibitory effects of the PDE4 inhibitors roflumilast and cilomilast related to respiratory diseases in PDE4. The crystal structures of PDE4 in complex with roflumilast and cilomilast are also analyzed. This review provides useful information for the future design of novel PDE4 inhibitors.

## 1. Introduction

Phosphodiesterases (PDEs) are multigene superfamily enzymes responsible for the hydrolysis of cyclic adenosine monophosphate (cAMP) and cyclic guanosine monophosphate (cGMP) into AMP and GMP, respectively [1]. These cyclic nucleotides act as essential secondary messengers, modulating a wide range of physiological processes, including gene expression, metabolism, and cell proliferation [2]. The regulation of intracellular cAMP and cGMP levels by PDEs ensures the precise control of various signaling pathways, thereby maintaining cellular homeostasis [3,4,5].

Among the 11 identified PDEs, PDE4 was the first discovered variant and specifically hydrolyzes cAMP, thereby regulating the intracellular concentration of this critical secondary messenger [6]. Four distinct genes—PDE4A, PDE4B, PDE4C, and PDE4D—encode the PDE4 isoforms, which share a highly conserved catalytic domain but exhibit functional diversity due to alternative splicing and different regulatory elements. These isoforms are classified into long, short, super-short, and dead-short forms based on the presence and length of two upstream conserved regions (UCR1 and UCR2), which modulate enzymatic activity through post-translational modifications, notably phosphorylation by protein kinase A (PKA) and extracellular signal-regulated kinase (ERK) [7,8,9].

**Figure 1:**
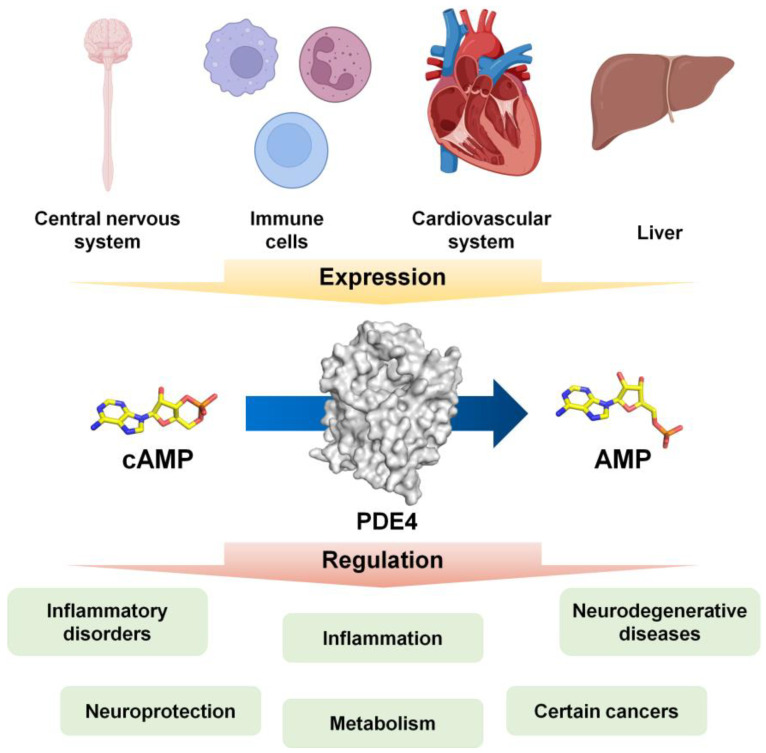
Overview of PDE4 expression and regulation. PDE4 is expressed in various tissues and cell types. Inhibition of PDE4 leads to the accumulation of cyclic adenosine monophosphate (cAMP) by preventing its degradation, which in turn modulates the pathophysiology of inflammatory diseases, metabolic disorders, cancer, and neurological conditions. The images were obtained from BioRender (https://biorender.com/: accessed on 22 November 2024).

PDE4 isoforms are widely expressed across various cells, tissues, and organs, including the central nervous system (CNS), immune cells, the cardiovascular system, and the liver, where they regulate essential physiological processes such as inflammation, neuroprotection, and metabolism [9,10,11]. Dysregulation of PDE4 activity has been implicated in numerous pathological conditions, including neurodegenerative diseases, inflammatory disorders, and certain cancers, making PDE4 a promising therapeutic target [10]. PDE4 is considered a versatile pharmacological target, and its inhibitors are being explored for the treatment of cystic fibrosis, multiple sclerosis, CNS disorders such as depression, and cancer, where they modulate inflammation and immune responses [12,13,14]. Consequently, there is ongoing research not only into the in-depth functional understanding of PDE4 but also into the development of its inhibitors [15,16,17,18] (see Figure 1).

This review provides a comprehensive analysis of the molecular characteristics of human PDE4, focusing on its structural and functional attributes. It further examines the inhibitory mechanisms of roflumilast and cilomilast, elucidating their molecular interactions with PDE4 and exploring their therapeutic applications in various diseases. By compiling current insights into PDE4 inhibition, this review highlights the clinical potential and challenges associated with PDE4-targeted drug development.

## 2. Disease-Related Biological Functions of PDE4

The regulation of cAMP levels by PDE4 influences various physiological and pathological processes. In particular, the biological function of PDE4 is related to conditions such as inflammatory diseases, neurodegenerative disorders, and hepatic pathophysiology.

### 2.1. Inflammatory Diseases

PDE4 plays a central role in modulating inflammatory responses by regulating cAMP levels in immune cells, including T lymphocytes, macrophages, and dendritic cells. Elevated PDE4 activity leads to reduced intracellular cAMP levels, which enhances the production of proinflammatory cytokines such as TNF-α, IL-6, and IL-1β [19]. This mechanism is particularly relevant in the context of chronic inflammatory diseases such as chronic obstructive pulmonary disorder (COPD) and asthma. In respiratory inflammation, PDE4 isoforms contribute to airway hyperresponsiveness and tissue remodeling [20,21].

### 2.2. Neurodegenerative Disorders

In the CNS, PDE4 is highly expressed in regions such as the hippocampus and striatum, where it regulates cognitive processes, memory consolidation, and synaptic plasticity [22]. Dysregulation of PDE4 activity has been linked to neurodegenerative diseases, including Alzheimer’s disease (AD), Parkinson’s disease (PD), and Huntington’s disease (HD), primarily through the disruption of cAMP/PKA/CREB signaling [23]. For example, reduced cAMP levels in neurodegenerative conditions contribute to neuroinflammation and neuronal apoptosis. Specific isoforms, such as PDE4D4 and PDE4D6, are particularly implicated in these processes [24,25].

### 2.3. Hepatic Pathophysiology

In hepatic tissues, PDE4 modulates key processes such as inflammation, fibrosis, and apoptosis through cAMP-dependent pathways. Dysregulated PDE4 activity in hepatic stellate cells contributes to fibrogenesis, which is a hallmark of chronic liver diseases such as hepatitis and non-alcoholic steatohepatitis (NASH) [26].

## 3. PDE4 Subfamily

The human genome contains 21 PDE genes, which are classified into 11 families (PDE1–PDE11) [27,28]. In addition, alternative splicing of PDE mRNAs generates 120 or more distinct PDE isoforms in various human tissues [27]. These PDE isoforms generally contain a catalytic domain, and the alternative splicing of these PDE domains alters phosphorylation, oligomerization, cellular localization, and interactions with other proteins, leading to functional diversity [27]. The domain organization of the PDE4 subfamily has been reported in previous studies. To better understand the diversity of the PDE4 subfamily and its isoforms, as well as their potential therapeutic applications, we performed a detailed analysis of them using data from the past literature and the UniProt protein database (https://www.uniprot.org/; accessed on 9 August 2024).

**Figure 2:**
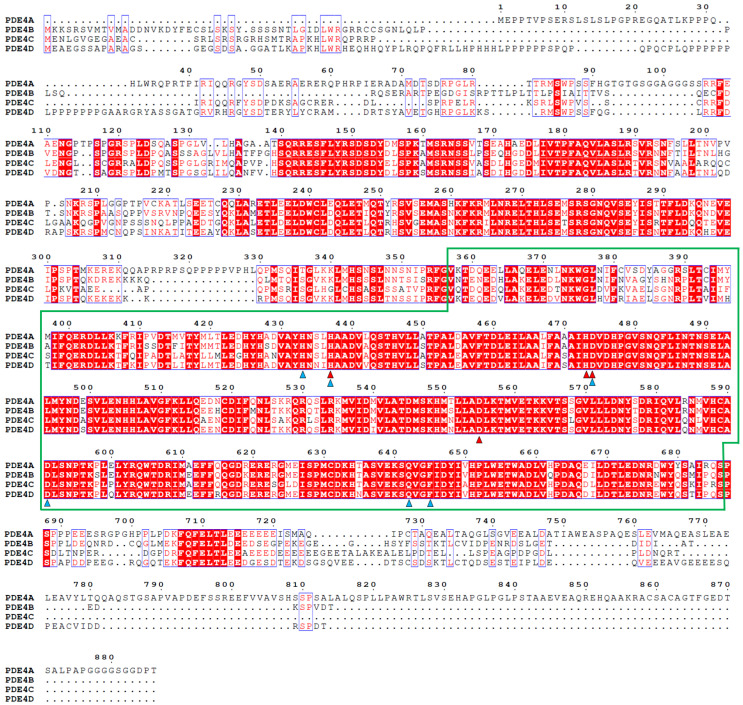
Alignment of the canonical amino acid sequences of PDE4A (UniProt code: P27815), PDE4B (Q07343), PDE4C (Q08493), and PDE4D (Q08499). The catalytic domain region of the PDE4A subfamily is indicated by a green box. The “HD” domain motif for Mg^2+^ (or Mn^2+^) and Zn^2+^ binding is indicated by red triangles, while the 3′,5′-cyclic AMP (or AMP) binding site is indicated by blue triangles.

The human PDE4 family is composed of four subfamilies (PDE4A, PDE4B, PDE4C, and PDE4D) that are independently encoded by different genes [29,30]. The canonical PDE4A, PDE4B, PDE4C, and PDE4D consist of 886, 736, 712, and 809 amino acids, respectively. Although the numbers of amino acids vary between the PDE4 subfamily, the amino acid sequences of all of the PDE4 catalytic domains show high identity (Figure 2). The numbers of amino acids of the catalytic domains (330 amino acids, Val357–Ser686: numbered in PDE4A) in the PDE4 subfamily were also found to be identical. The sequence alignment of the catalytic domains among the canonical PDE4 subfamilies showed sequence identities of 82.4–87.6%. All of these PDE4s have a conserved “HD (histidine–aspartate)” motif consisting of His437, His473, Asp474, and Asp555 (numbered in PDE4A) of the phosphohydrolase domain, which is involved in Mg^2+^ and Zn^2+^ binding and the regulation of enzymatic activity and is essential for the hydrolysis of cAMP by PDE4 [31]. Moreover, the cAMP (or AMP) binding residues, including His433, His437, Asp474, Asp591, Gln642, and Phe645, were also found to be conserved among the PDE4 subfamilies (Figure 2).

The multiple upstream exons of the four PDE4 subtype genes undergo complex splicing, generating multiple PDE4 isoforms [32,33]. These PDE4 isoforms are targeted to different cellular microdomains and involved in the isoform- and subtype-selective regulation of PDE4 activity by accessory proteins [33,34,35], having different expression levels and molecular functions in tissues and cells [36]. According to the UniProt database, the PDE4A, PDE4B, PDE4C, and PDE4D groups comprise 7, 4, 7, and 12 isoforms, respectively. Detailed information on PDE4 isoforms obtained from ExPASy is summarized in Table S1.

**Figure 3:**
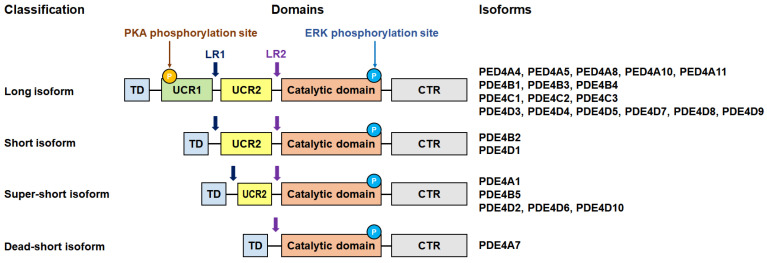
PDE4 isoform structure and classification. This is an adapted version of figures in a previous report [37] and has been modified. The information on isoform classification was obtained from a previous study [39]. The subclasses of PDE4 are categorized depending on the presence of the UCR in the long, short, super-short, and dead-short isoforms. TD, transduction domain; LR, linker region; UCR, upstream conserved region; CTR: C-terminal region.

The four subfamilies of PDE4 possess an upstream conserved region (UCR) that plays a key role in controlling intracellular signaling [22] (Figure 3). Based on the presence of UCRs, PDE4 isoforms are further categorized into subclasses, namely, long, short, super-short, and dead-short variants [37]. The long isoforms include both UCR1 and UCR2, the short isoforms contain only UCR2, and the super-short isoforms have a truncated version of UCR2 [38]. In addition, dead-short isoforms were also reported to be present in PDE4A7, which lacks the UCR2 region [37]. The regulation of PDE4 is mediated through phosphorylation by protein kinase A (PKA) and extracellular signal-regulated kinase (ERK), highlighting the critical functional roles of the UCR modules [9]. UCR1 serves as a phosphorylation site for PKA, enhancing the activity of long isoforms and facilitating the localized control of cAMP levels [9]. ERK phosphorylates all PDE4 isoforms, but its effect on PDE4 activity varies depending on the presence of UCR1. While ERK phosphorylation suppresses the activity of the long isoforms, it enhances that of the short isoforms and has no effect on the super-short isoforms [9].

For PDE4A, the canonical PDE4A1 consists of 886 amino acids, while the lengths of other PDE4A isoforms (PDE4A2–7) vary from 323 to 864 amino acids (Table S1). The subcellular locations of these PDE4A isoforms also vary. For example, PDE4A1 is known to be located in the cytoplasm and perinuclear region, whereas PDE4A4 and PDE4A7 are located in the membrane and cytosol, respectively. Based on the protein database, analysis of canonical PDE4A showed that the catalytic domain consists of 330 amino acids (Val357–Ser686), which includes the active site (His433; proton donor), the 3′,5′-cyclic AMP-binding site (His433, Gln642, Phe645), the AMP-binding site (His433, His437, Asp474, Asp591, Gln642, Phe645), and the metal binding site (His437, His473, Asp474, Asp591 for Zn^2+^; Asp474 for Mg^2+^ [or Mn^2+^]). Although the PDE4A isoforms exhibit variations in amino acid composition and subcellular location, the length and sequence of the catalytic domain of the canonical PDE4A1 are identical to those of PDE4A2, PDE4A3, PDE4A4, PDE4A6, and PDE4A7 (Supplementary Figure S1). Meanwhile, the N- and C-terminal amino acid sequences of PDE4A5 differ from those of the canonical PDE4A1, but the functional amino acid sequences involved in the catalytic site, the 3′,5′-cyclic AMP-binding site, the AMP-binding site, and the metal binding site are conserved (Supplementary Figure S1).

For PDE4B, the canonical PDE4B1 consists of 736 amino acids, while the PDE4B2, PDE4B3, and PDE4B4 isoforms consist of 564, 721, and 503 amino acids, respectively (Table S1). Based on the protein database, analysis of canonical PDE4B showed that the catalytic domain consists of 330 amino acids (Val330–Ser659), which includes the active site (His406; proton donor), the 3′,5′-cyclic AMP-binding site (His406, Gln615, and Phe618), the AMP-binding site (His406, His410, Asp447, Asp564, Gln615, and Phe618), and the metal binding site (His410, His446, Asp447, and Asp564 for Zn^2+^; Asp447 for Mg^2+^; Asp447 for Mn^2+^). Although the N-terminal region of the PDE4A isoforms exhibited variations in amino acid composition, the catalytic domain of the canonical PDE4B1 was absolutely conserved relative to the other PDE4B isoforms (Supplementary Figure S2).

For PDE4C, seven PDE4C isoforms have been classified in the protein database, but only the amino acid sequences of three of them (PDE4C1–3) are available (https://www.uniprot.org/uniprotkb/Q08493/entry; accessed on 9 August 2024). The canonical PDE4C1 consists of 712 amino acids, while the PDE4C2 and PDE4C3 isoforms consist of 606 and 680, respectively (Table S1). Based on the protein database, analysis of canonical PDE4B showed that the catalytic domain consists of 330 amino acids (Val312–Ser641), which includes the active site (His388; proton donor), the 3′,5′-cyclic AMP-binding site (His388, Gln597, and Phe600), the AMP-binding site (His388, His392, Asp429, Asp546, Gln597, and Phe600), and the metal binding site (His392, His428, Asp429, Asp546 for Zn^2+^; Asp429 for Mg^2+^; Asp 429 for Mn^2+^). Sequence alignment showed that the 606 amino acids (Met107–Thr712) containing the catalytic domains of PDE4C1, PDE4C2, and PDE4C3 were completely conserved, whereas the N-terminus of these isoforms varied (Supplementary Figure S3).

For PDE4D, canonical PDE4D4 consists of 809 amino acids, while the other PDE4D isoforms vary in length from 215 to 748 amino acids (Table S1). Based on the protein database, analysis of the canonical PDE4D showed that the catalytic domain consists of 330 amino acids (Val386–Ser715), which includes the active site (His462; proton donor), the 3′,5′-cyclic AMP-binding site (His462, Gln671, and Phe674), the AMP-binding site (His462, Asp503, Asp620, Asn623, Gln671, and Phe674), and the metal binding site (His466, His502, Asp503, and Asp620 for Zn^2+^; Asp503 for Mg^2+^; Asp503 for Mn^2+^). Sequence alignment showed that the 502 amino acids (Lys307–Thr809) containing the catalytic domain of canonical PDE4D4 were conserved, with hPDE4D3, hPDE4D1, hPDE4D2, hPDE4D5, PDE4DN3, PDE4D6, PDE4D8, PDE4D9, and PDE4D7 being completely conserved, whereas the N-terminus of these isoforms varied (Supplementary Figure S4). Meanwhile, PDE4DN3 and PDE4D12 have no catalytic domains, indicating that these two isoforms do not exert enzymatic activity.

## 4. Structure of the PDE4s

### 4.1. Overall Structure of PDE4

To date, 5 PDE4A, 40 PDE4B, 2 PDE4C, and 110 PDE4D structures have been deposited in the Protein Data Bank (PDB; https://www.rcsb.org/) (accessed on 22 November 2024) (Table S2). All PDE4 structures were determined by X-ray crystallography, apart from one NMR structure of PDE4D (PDB code: 1E9K). These experimentally determined PDE4 structures were constructed with only the catalytic domain region, but without other regions, indicating that the full-length PDE4 structure has not yet been determined. To understand the architecture of full-length PDE4A–D, the model structures of canonical PDE4A, PDE4B, PDE4C, and PDE4D were generated using AlphaFold3 [40] (Supplementary Figure S5). The model structures of PDE4A, PDE4B, PDE4C, and PDE4D showed that the conserved regions comprising the catalytic domains of PDE4s consist of an α-helical bundle, while the non-conserved regions are composed mainly of loop regions and some α-helices. The loop regions in the modeling structures of the full-length PDE4s have low predicted local distance difference test (pLDDT) scores, a per-residue measure of local confidence, so they do not provide convincing structural and functional information. Accordingly, we describe only the catalytic domain of PDE4 based on the previously determined structures and the literature.

**Figure 4:**
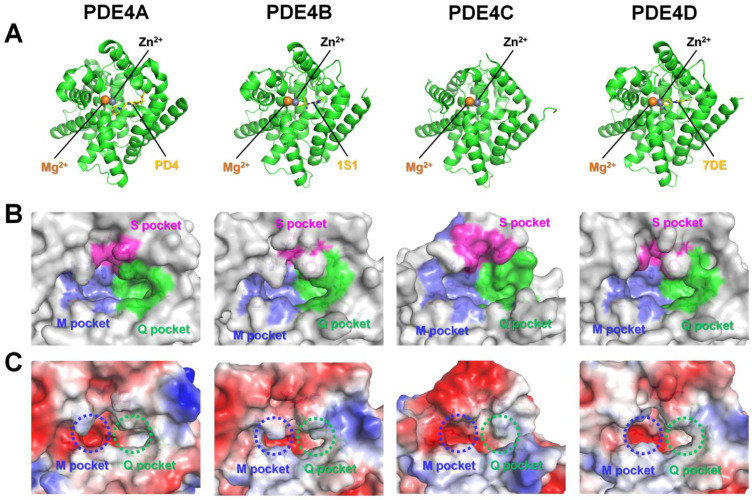
Crystal structures of the catalytic domain of PDE4s. (**A**) Cartoon representation of the catalytic domains of PDE4A (PDB code: 3HC8), PDE4B (4KP6), PDE4C (2QYM), and PDE4D (1Y2K). (**B**) Surface and (**C**) electrostatistic surface representation of the active site pockets of PDE4A–PDE4D. The metal binding pocket, Q switch and P clamp pocket, and solvent-filled side pocket are indicated by yellow, green, and magenta, respectively.

The catalytic domains of PDE4A, PDE4B, PDE4C, and PDE4D consist of 16–17 α-helices, showing mostly similar folding (Figure 4A). The catalytic domains of the PDE4 family can be divided into three subdomains, which form deep pockets containing the active site of PDE4s where these subdomains meet (Figure 4B).

In a previous study, the substrate binding pocket of PDE4 was divided into three functional pockets [41]: a metal binding pocket (M pocket), a Q switch and P clamp pocket (Q pocket), and a solvent-filled side pocket (S pocket) (Figure 4B). The M pocket contains two divalent metal ions, Zn^2+^ and either Mg^2+^ or Mn^2+^, which are essential for substrate hydrolysis. The Q pocket includes a conserved glutamine residue and the P clamp, flanked by two narrow and deep hydrophobic subpockets (Q1 and Q2). Notably, the Q pocket showed less conservation across the PDE family, suggesting that this region may be a worthwhile target for developing isoform-selective PDE inhibitors [41]. The S pocket is primarily composed of hydrophilic residues and is filled with water molecules that form a stabilizing network.

In this previous study, key residues involved in the M, Q, and S pockets of PDE4B and PDE4D were analyzed [41], exhibiting identical amino acids in PDE4B and PDE4D contributing to the substrate binding pocket at the same positions in their 3D structures (Table S3). This analysis showed that the M, Q, and S pockets of PDE4B and PDE4D consist of 11, 14, and 7 amino acids, respectively. This study classified that residues Tyr403, Trp406, and Ser442 (numbered as in PDE4B) are involved in the formation of the Q pocket [41]. However, in our structural analysis, these residues were located on the opposite side of the Q pocket surface (Supplementary Figure S6), indicating that they do not directly contribute to the Q pocket in the active site, contrary to previous reports. Additionally, Asn283, Asp346, and Met347 (numbered as in PDE4B) were previously classified as residues forming the M pocket; however, in our analysis, these residues were positioned near the entrance of the substrate binding pocket without interacting with metal ions, such as Zn^2+^ or Mg^2+^ (Supplementary Figure S7), indicating that these residues are not involved in the M pocket. Accordingly, the key residues involved in the Q and M pockets within the active sites of PDE4B and PDE4D will be reassigned.

The position and distribution of the residues involved in the Q, M, and S pockets of PDE4A–D were similar (Figure 4B), but the detailed architecture differed due to the different ligands bound to the active sites of PDE4A–D. For example, in the selected high-resolution model structure, PDE4A, PDE4B, and PDE4D were complexed with Mg^2+^, Zn^2+^, and inhibitors, but the chemical structure of the inhibitor differed, inducing a different conformation of the entrance of the substrate binding pocket due to their unique ligand interaction. Meanwhile, PDE4C was complexed with Mg^2+^ and Zn^2+^, without the chemical compound, showing the disordered S pocket region and not showing the deeper substrate binding pocket in the crystal structure. Because the crystal structure of identical ligand-bound PDE4s was not reported, the formation of the substrate binding pocket of PDE4A–4D could not be compared directly. Meanwhile, in the electrostatic surface, the M and Q pockets of PDE4A–4D commonly have negative and hydrophobic charged surfaces, respectively, indicating that the overall ligand recognition in the active site pocket of PDE4s is similar (Figure 4C).

**Figure 5:**
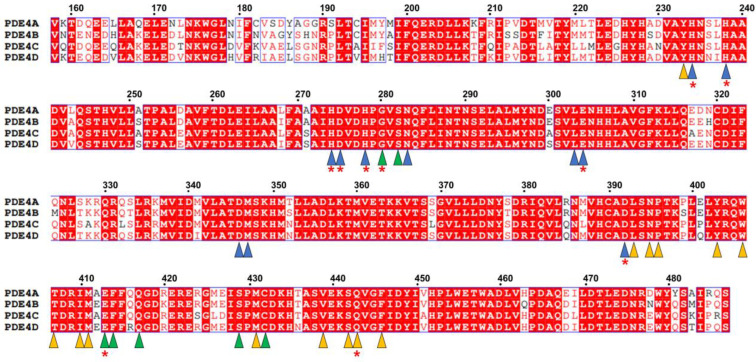
Partial sequence alignment of the catalytic domain of the canonical PDE4 subfamily (numbered as in PDE4B). The residues involved in the metal binding pocket, Q switch and P clamp pocket, and solvent-filled side pocket are indicated by blue, yellow, and green triangles. The three functional binding pockets of PDE4B and PDE4C were classified in a previous study [41]. Residues that are completely conserved in all PDEs are indicated by red asterisks.

In a previous study, the residues involved in the substrate binding pockets of PDE4B and PDE4D were classified [41]; however, the substrate binding pockets of PDE4A and PDE4C have not yet been classified in detail into three categories. Sequence alignment of the catalytic domain showed that the key residues involved in the active site pockets of PDE4B and PDE4D are conserved across the PDE4 subfamily (Figure 5), providing further analysis of the substrate binding pockets of PDE4A and PDE4C.

### 4.2. Metal Binding Site and Substrate Recognition of PDE4B and PDE4D

**Figure 6:**
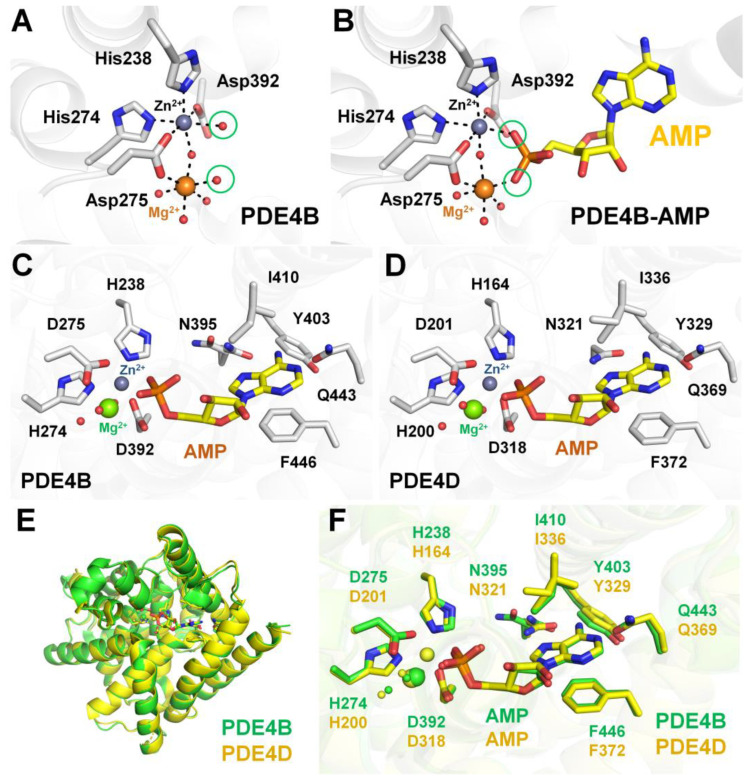
Analysis of metal ion coordination and AMP binding in PDE4B and PDE4D. (**A**) Coordination of Zn^2+^ and Mg^2+^ in native PDE4B (PDB code: 1TAZ). (**B**) AMP binding in the PDE4B complex (PDB code: 1TB5) with metal ions. AMP binding in the (**C**) PDE4B (PDB code: 1TB5) and (**D**) PDE4D complex (PDB code: 1TB7) with metal ions. (**E**) Superimposition of the AMP-bound catalytic domains of PDE4B (green) and PDE4D (yellow). (**F**) Close-up view of the AMP-binding site in PDE4B (green) and PDE4D (yellow).

To better understand substrate cAMP recognition, we here reviewed the previously determined crystal structures of Zn^2+^- and Mn^2+^-bound PDE4B (PDB code: 1F0J) [42] and the AMP-, Zn^2+^-, and Mn^2+^-bound catalytic domains of PDE4B (1TB5) and PDE4D (1TB7) [43]. In Zn^2+^- and Mn^2+^-bound PDE4B, Zn^2+^ and Mg^2+^ are positioned at the binuclear metal ion center at the M pocket (Figure 6A). Zn^2+^ is coordinated by two conserved histidines (His238 and His274), two aspartic acids (Asp275 and Asp392), and one water molecule. Mg^2+^ is coordinated by the conserved Asp275 and four water molecules. These two metal ions are bridged by the conserved Asp275 and a water molecule (Figure 6A). The position of these metal ions and their interaction with conserved residues are identical to those of other Zn^2+^- and Mg^2+^-bound PDE4s, but the water coordination differs (see below). For example, in the crystal structure of PDE1B (1TAZ), the Zn^2+^ is octahedrally coordinated by two conserved histidines (His227 and His263), two aspartic acids (Asp264 and Asp370), and two water molecules. Mg^2+^ is also octahedrally coordinated by the conserved Asp264 and five water molecules (Figure 6A).

In the AMP-, Zn^2+^-, and Mn^2+^-bound catalytic domain of PDE4B, AMP, the product of the hydrolytic reaction, binds to both the M and Q pockets at the active site (Figure 6B). The phosphate group of AMP coordinates with Zn^2+^ and Mg^2+^ in the M pocket of PDE4B, forming the octahedral coordination of the Zn^2+^ and Mg^2+^. The adenosine base of AMP occupies the Q pocket, while the ribose group of AMP is positioned between the M and Q pockets (Figure 6B). The completely conserved Gln443 forms two hydrogen bonds with the adenine moiety of AMP. The orientation of Gln443 is stabilized by a hydrogen bond with the hydroxyl group of Tyr403, and the adenine ring additionally forms a hydrogen bond with Asn395 (Figure 6B). The binding mode of AMP in PDE4D was found to be nearly identical to that in PDE4B (Figure 6C). Therefore, two water molecules involved in the coordination of the Mg^2+^ or Zn^2+^ at the binuclear metal ion center at the M pocket of native PDE4B/PDE4D alternated with two oxygen atoms in the phosphate group of the AMP, showing the stable coordination of the binuclear metal ion center of PDE4B/PDE4D (Figure 6D). These structural results indicate that the binding and selectivity of the cAMP substrate in PDE4B and PDE4D require various interactions, including metal binding, phosphate group coordination, hydrophobic affinity, and hydrogen bonding with the nucleotide [43]. Superimposition of AMP-, Zn^2+^-, and Mg^2+^-bound PDE4B and PDE4D structures showed high similarity, with an r.m.s. deviation of 0.498 Å (Figure 6E). The position and conformation of the residues involved in AMP coordination were almost identical, except for the residue between Asn395 from PDE4B and Asn321 from PDE4D.

## 5. Biological Roles of PDE4 Inhibitors in Disease

**Figure 7:**
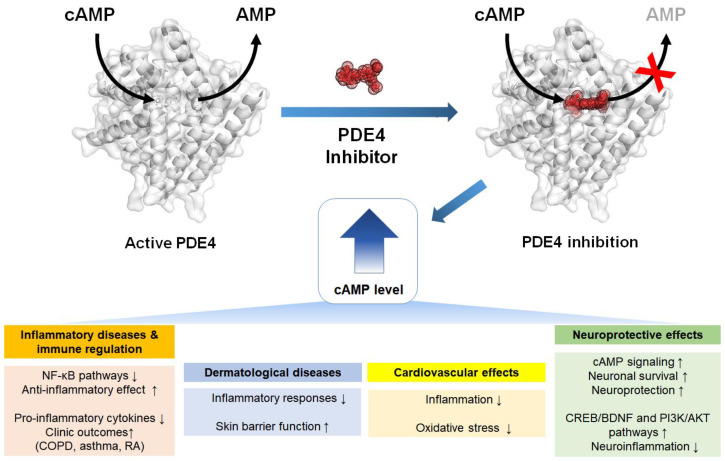
PDE4 inhibition elevates the cAMP level, affecting various signaling pathways such as those involved in inflammatory diseases and immune regulation, neuroprotective effects, dermatological diseases, and cardiovascular effects. COPD = chronic obstructive pulmonary disease, CREB = cAMP response element-binding protein, BDNF = brain-derived neurotrophic factor.

The inhibition of PDE4 has diverse effects by elevating the levels of cyclic adenosine monophosphate (cAMP), which subsequently regulates a wide array of genes and proteins [44] (Figure 7). Inhibitors of PDE4 may be useful for modulating the gene expression of proinflammatory and anti-inflammatory cytokines, either positively or negatively [45]. Accordingly, PDE4 inhibition is considered beneficial as a treatment for various diseases.

### 5.1. Inflammatory Diseases and Immune Regulation

Selective PDE4 inhibitors, such as roflumilast and cilomilast, elevate intracellular cAMP levels, exerting potent anti-inflammatory effects by inhibiting the NF-κB and MAPK pathways. These inhibitors suppress the production of proinflammatory cytokines, thereby improving clinical outcomes in diseases such as chronic obstructive pulmonary disorder, asthma, and rheumatoid arthritis (RA) [46,47,48,49]. Meanwhile, roflumilast has shown efficacy in reducing inflammation and improving lung function in a murine model of asthma [20].

### 5.2. Neuroprotective Effects

In models of neurodegeneration, PDE4 inhibitors enhance cAMP signaling, promoting neuronal survival and neuroprotection. For example, roflumilast improves motor function and reduces neuroinflammation in PD models by activating the CREB/BDNF and PI3K/AKT pathways [23]. Moreover, in spinal cord injury (SCI), roflumilast facilitates functional recovery by reducing apoptosis and enhancing neuronal differentiation [25,50].

### 5.3. Dermatological Diseases

The PDE4 inhibitor roflumilast is used in treating dermatological conditions such as skin fibrosis, including keloid and hypertrophic scars. By restoring cAMP levels via the inhibition of PDE4B, the PDE4 inhibitors attenuate inflammatory responses and improve skin barrier function [51]. Inhibition of PDE4 prevented TGFβ1-induced Smad3 and ERK1/2 phosphorylation and myofibroblast differentiation, reducing the activation of dermal fibroblasts [51].

### 5.4. Cardiovascular Effects

PDE4 inhibitors have shown protective effects against cardiovascular diseases, including sepsis-induced cardiovascular dysfunction and myocardial injury. By increasing cAMP levels, these inhibitors reduce inflammation and oxidative stress, although caution is required regarding their clinical use due to potential side effects [21,52,53]. Roflumilast prevents NO-induced apoptosis through both cAMP–PKA/CREB and Epac/Akt signaling pathways, suggesting its potential as a therapeutic agent for myocardial ischemia/reperfusion injury [21].

**Figure 8:**
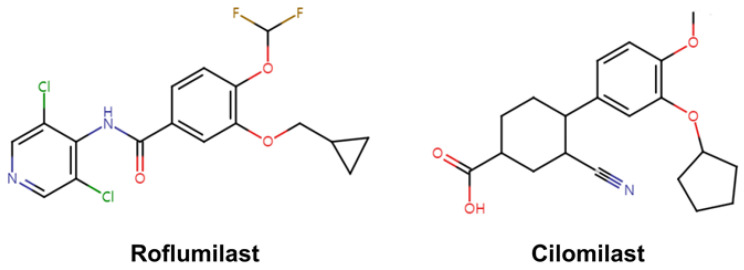
Chemical structures of roflumilast and cilomilast.

Numerous PDE4 inhibitors have been developed [44,54,55,56]. To date, roflumilast, apremilast, and crisaborole have been approved for the treatment of inflammatory airway diseases, psoriatic arthritis, and atopic dermatitis, respectively [44]. Among these various potential PDE4 inhibitors, the second generation of selective PDE4 inhibitors, such as roflumilast and cilomilast (Figure 8), have demonstrated clinical efficacy by elevating intracellular cAMP levels, thereby suppressing proinflammatory cytokine production and modulating inflammatory responses [57,58,59,60,61]. In particular, roflumilast, approved by the US Food and Drug Administration for treating chronic obstructive pulmonary disorder, exerts significant anti-inflammatory effects, while cilomilast has shown potential in preclinical and clinical studies for its anti-inflammatory and bronchodilatory properties [46].

Various biochemical studies have examined the inhibitory effects of roflumilast and cilomilast against members of the PDE family [62]. For example, Hatzelmann and Schudt investigated the inhibitory effects of these compounds and showed that roflumilast achieved significant inhibition of PDE4, with a half-maximal inhibitory concentration (IC_50_) value of 0.8 nM, whereas it exhibited low inhibition effects on PDE1 (bovine brain: IC_50_ > 10 μM), PDE2 (rat heart: IC_50_ > 10 μM), PDE3 (human platelets: IC_50_ > 10 μM), or PDE5 (human platelets: IC_50_ = 8 μM). Meanwhile, cilomilast exhibited inhibition of PDE4 with an IC_50_ of 120 nM but had no effect on PDE1 (74 μM), PDE2 (65 μM), PDE3 (>1000 μM), or PDE5 (83 μM) [63]. Card et al. also conducted a biochemical inhibition study on roflumilast and cilomilast [41]. In that study, roflumilast inhibited PDE4B and PDE4D with IC_50_ values of 8.4 and 6.8 nM, respectively, while showing low inhibition of PDE1B (>200 μM), PDE2A (>200 μM), PDE3B (>200 μM), PDE5A (17 μM), PDE7B (>200 μM), PDE8A (>200 μM), PDE10A (>200 μM), and PDE11A (25 μM). Moreover, cilomilast inhibited PDE4B and PDE4D with IC_50_ values of 25 and 11 nM, respectively, while showing low inhibition of PDE1B (87 μM), PDE2A (160 μM), PDE3B (87 μM), PDE5A (53 μM), PDE7B (44 μM), PDE8A (7 μM), PDE10A (73 μM), and PDE11A (21 μM). While the determined IC_50_ values differ slightly among research groups, both roflumilast and cilomilast exhibited significant inhibition of PDE4, with minimal effect on other PDEs, indicating their potential as valuable candidates for drug design.

## 6. Crystal Structure of PDE4 Complexed with Roflumilast and Cilomilast

All known PDE inhibitors bind primarily around the Q pocket and occasionally near the M pocket (Figure 5). These inhibitors are stabilized through three major types of interactions: (i) hydrophobic interactions with residues lining the active site pocket, (ii) hydrogen bonding with nucleotide-recognizing residues, and (iii) water-mediated interactions with metal ions [41]. Among the various PDE4 inhibitors, roflumilast and cilomilast have been identified as high-potency inhibitors of PDE4B and PDE4D and have advanced to Phase III clinical trials [64]. To date, the crystal structures of roflumilast-bound PDE4B (PDB code: 1XMU) and PDE4D (PDB codes: 1XQQ and 3G4L), as well as cilomilast-bound PDE4B (PDB code: 1XLX) and PDE4D (PDB codes: 1XQQ and 1XOM), have been determined (Table 1). Here, we review not only the previously reported binding properties of inhibitors to each PDE4 but also analyze the structural features not described in previous studies to provide insights for future inhibitor development.

### 6.1. Roflumilast-Bound State of PDE4B and PDE4D

**Figure 9:**
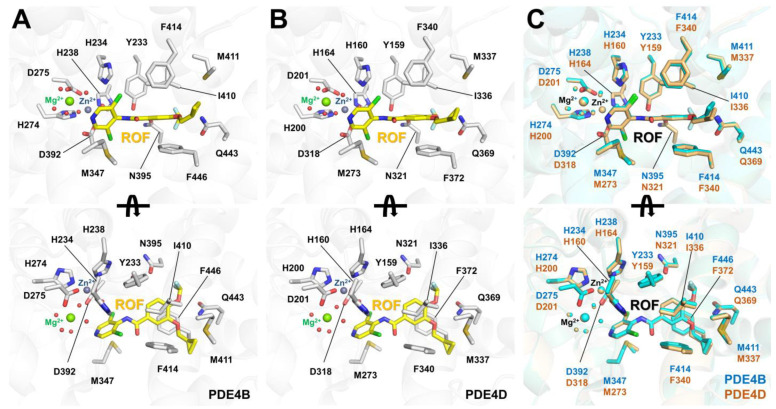
Crystal structures of PDB4 complexed with the roflumilast. Close-up view of roflumilast (ROF) binding to (**A**) PDE4B (PDB code: 1XMU) and (**B**) PDE4D (PDB code: 1XOQ). (**C**) Superimposition of the roflumilast-bound PDE4B (cyan) and PDE4D (light orange) structures.

In roflumilast-bound PDE4B and PDE4D, the M pocket is occupied by Zn^2+^ and Mg^2+^ ions [41,65]. These metal ions are octahedrally coordinated with conserved histidine and aspartic acid residues, as well as a water molecule. However, the position of the metal ions and the water molecule and the distances between the metal ions and their coordinating residues differ slightly between roflumilast-bound PDE4B (PDB code: 1XMU) and PDE4D (1XOQ and 3G4L) (see below). This indicates that the M pocket, containing the metal ions and water molecule, is not rigid. The overall binding configurations of the roflumilast at the active site pocket of PDE4B and PDE4D were similar (Figure 9A,B). The dichloropyridyl group of roflumilast is positioned in the M pocket of PDE4s, while the difluoromethoxy and cyclopropylmethyl groups are located in the Q pocket. The nitrogen atom in the dichloropyridyl group of roflumilast forms a hydrogen bond with a water molecule coordinated with Mg^2+^, with distances of 3.01/3.13 Å and 2.89/2.93 Å for PDE4B-1XMU and PDE4D-1XOQ, respectively [41]. In PDE4D-3G4L at 2.5 Å resolution, the distance between roflumilast and the water molecule interacting with Mg^2+^ across three PDE4D molecules ranges from 2.63 to 3.04 Å, while no water molecule interacting with Mg^2+^ is observed in one other PDE4D molecule [65]. The dialkoxyphenyl group is considered a scaffold for various inhibitors of PDE4B and PDE4D. The dialkoxyphenyl group of roflumilast is sandwiched between the hydrophobic clamp, with Phe446 and Ile410 in PDE4B and Phe372 and Ile336 in PDE4D (Figure 9B). In PDE4B, the difluoromethoxy group of roflumilast binds at the Q1 pocket, and its fluoride atoms and oxygen atom interact with the OD1 atom of Asn395 and the NE2 atom of Gln443, respectively, at distances of 3.43–3.75 Å and 3.03–3.16 Å. The cyclopropyl methyl group of roflumilast is located at the Q2 pocket and stabilized by hydrophobic residues Met411, Phe414, Met431, and Phe446. In PDE4D, the difluoromethoxy group of roflumilast binds at the Q1 pocket, and its fluoride atoms and oxygen atom interact with the OD1 atom of Asn395 and the NE2 atom of Gln369, respectively, at distances of 3.43–3.67 Å and 3.08–3.15 Å. The cyclopropyl methyl group of roflumilast is located at the Q2 pocket and stabilized by hydrophobic residues Met337, Phe340, Met357, and Phe372 [41]. Therefore, the binding modes of roflumilast to PDE4B and PDE4D exhibit high similarity.

The superimposition of roflumilast-bound PDE4B and PDE4D showed an r.m.s. deviation of 0.241–0.295 Å (Figure 9C). The positioning of roflumilast in PDE4B and PDE4D is nearly identical, except for a subtle shift in the cyclopropyl methyl group in one of the PDE4D molecules. This structural analysis suggests that the binding modes and binding affinities of roflumilast in PDE4B and PDE4D are highly similar. This observation is consistent with previous biochemical results, which reported IC_50_ values for roflumilast of 0.00084 μM for PDE4B and 0.00068 μM for PDE4D [41]. Accordingly, novel roflumilast-based inhibitors may be suitable for targeting both PDE4B and PDE4D.

### 6.2. Cilomilast-Bound State of PDE4B and PDE4D

In cilomilast-bound PDE4B (PDB code: 1XLX) and PDE4D (PDB code: 1XOM), the M pocket contains Zn^2+^ and Mg^2+^ ions, which are octahedrally coordinated with conserved histidine and aspartic acid residues, along with a water molecule [41], similar to the coordination of metal ions in roflumilast-bound PDE4B and PDE4D. The cilomilast molecule occupies the active site pocket of both PDE4B and PDE4D. Subtle differences in the positions of the metal ions and water molecules, as well as in the distances between the metal ions and their interacting molecules, were observed (see below).

**Figure 10:**
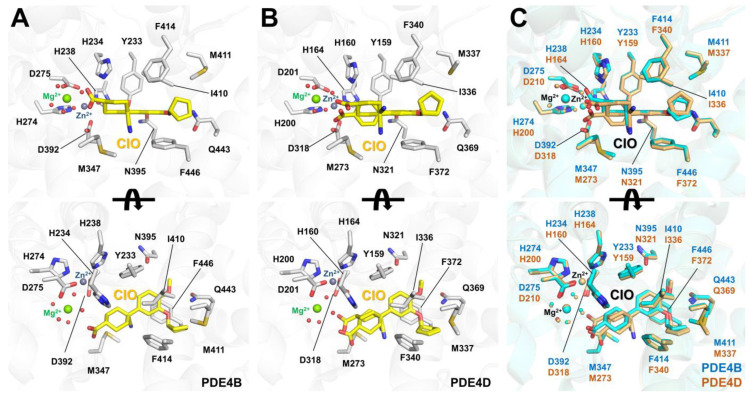
Crystal structures of PDB4 complexed with the cilomilast. (**A**) Close-up view of cilomilast binding to (**A**) PDE4B (PDB code: 1XLX) and (**B**) PDE4D (PDB code: 1XOM). In PDE4D, the carboxylcyclohexyl group of cilomilast exhibits two different conformations near the M pocket. (**C**) Superimposition of the cilomilast-bound PDE4B (cyan) and PDE4D (light orange) structures.

In PDE4B, the dialkoxyphenyl group of cilomilast is sandwiched between the hydrophobic clamp formed by Phe446 and Ile410 (Figure 10A). Two oxygen atoms in the dialkoxyphenyl group of cilomilast are stabilized by hydrogen interactions with the NE2 atom of Gln443, at distances of 3.00–3.66 Å. The methoxy group and cyclopentylether group of cilomilast bind at the Q1 and Q2 pockets, respectively. The cyclopentylether group of the cilomilast forms hydrophobic interactions with Met411, Phe414, Met431, and Phe446. The cyclohexyl group of the cilomilast forms hydrophobic interactions with Met347, Leu393, and Phe414, while the carboxylate oxygens form two hydrogen bonds with water molecules that coordinate Mg^2+^ in the M pocket [41]. Superimposing the two PDE4B-cilomilast molecules from the asymmetric unit revealed an r.m.s. deviation of 0.224 Å, indicating a slight difference in the position of the cilomilast molecules, particularly in the position of the cyclopentylether group and its interacting hydrophobic residues (Figure 10A).

In PDE4D, the dialkoxyphenyl group of cilomilast is similarly sandwiched between the hydrophobic clamp formed by Phe372 and Ile336 (Figure 10B). Two oxygen atoms in the dialkoxyphenyl group are stabilized by hydrogen interactions with the NE2 atom of Gln369 at distances of 3.02–3.23 Å. The methoxy and cyclopentylether groups of cilomilast bind at the Q1 and Q2 pockets, respectively, which is identical to their binding in PDE4B. The cyclopentylether group forms hydrophobic interactions with Met337, Phe340, Met357, and Phe372. Meanwhile, the carboxylcyclohexyl group of cilomilast exhibits two different conformations in the two PDE4D complexes from the asymmetric unit. One conformation of the carboxylcyclohexyl group is similar to that observed in the PDE4B complex, while the other conformation rotates approximately 60–70° towards the Zn^2+^ ion in the M pocket compared with the first conformation [41]. Superimposing the two PDE4D-cilomilast molecules from the asymmetric unit yields an r.m.s. deviation of 0.136 Å, showing the variations in the positions of the carboxylate oxygens of cilomilast (Figure 10B). However, all oxygen atoms of the carboxylate group of cilomilast form hydrogen bonds with the water molecule that coordinates Mg^2+^ in the M pocket.

The superimposition of cilomilast-bound PDE4B and PDE4D shows an r.m.s. deviation of 0.290–0.337 Å (Figure 10C). While the position of cilomilast in PDE4B and PDE4D is not identical, the conformation of the dialkoxyphenyl group in both complexes remains the same. However, different conformations are observed for the carboxylcyclohexyl and cyclopentylether groups of cilomilast. This suggests that the binding mode and binding affinity of cilomilast in the active site pockets of PDE4B and PDE4D are distinct. Based on the conformational flexibility observed, cilomilast binds more rigidly to PDE4B than to PDE4D. This structural analysis is consistent with previous biochemical results, indicating that the half-maximal inhibitory concentrations (IC_50_) of cilomilast for PDE4B and PDE4D are 0.025 μM and 1.1 μM, respectively [41]. To develop a cilomilast-based inhibitor with enhanced efficacy for PDE4D, chemical modifications to the carboxylcyclohexyl group may be an important consideration for tighter binding.

## 7. Discussion

Understanding the molecular properties of PDE4 and its inhibition can lead to the development of targeted therapies for inflammatory diseases, neurological disorders, and other conditions linked to dysregulated cAMP signaling.

Here, we review disease-associated PDE4 functions and provide a comprehensive analysis of the amino acid sequences and protein structures of members of the PDE4 family. We conducted alignment of amino acid sequences to obtain an intuitive understanding of PDE4 subfamilies and their respective isoforms. While the catalytic domains of different PDE4 subfamilies show high similarity, the alignment did not provide direct information about regions excluding the catalytic domain of PDE4s. Previous research has demonstrated that regions excluding the catalytic domain of PDE4 play key roles in phosphorylation, oligomerization, cellular localization, and protein–protein interactions, all of which contribute to functional diversity [28,66,67]. Nonetheless, accurately predicting specific molecular functions from these amino acid sequences alone remains a significant challenge. Additionally, structural analysis of full-length PDE4s using AlphaFold3 indicates that, apart from the catalytic domain and a few helices, most other regions are nonstructural loops with a low value of pLDDT. This highlights the need for future efforts to explore the functional roles of full-length isoforms beyond sequence data alone. 

To date, many crystal structures of PDE4 have been determined, with approximately 150 different compounds reported in complex with its catalytic domain (Table S2). This review discusses the inhibitory effects and structural analyses of roflumilast and cilomilast, both of which demonstrated strong inhibitory effects on PDE4. In addition to reviewing the previously noted binding properties of those inhibitors on PDE4, we analyzed the positions of the water molecules and the molecular flexibility of the amino acids of PDE4 near the active site, which were previously relatively overlooked. The obtained findings offer valuable insights into the binding mechanisms of these inhibitors and provide insight for the future development of novel inhibitors. Structural analysis of PDE4 in complex with roflumilast or cilomilast revealed that, while the two inhibitors bind to the catalytic domain of PDE4 in similar manners, their binding modes differ. Specifically, roflumilast binds rigidly to the catalytic domains of PDE4B and PDE4D, displaying high structural similarity in the M pocket, whereas slight positional variations in the inhibitor are observed in the Q pocket. In contrast, cilomilast binds tightly to the Q pocket of PDE4B and PDE4D but exhibits high flexibility in the M pocket. These findings suggest that leveraging the binding characteristics of both inhibitors could lead to the development of novel inhibitors with higher affinity for the PDE4 catalytic domain. In particular, the chemical scaffold of such inhibitors should be designed based on the structurally rigid framework of roflumilast. Furthermore, structural analysis of cilomilast binding to PDE4B and PDE4D indicated a potentially higher inhibitory effect on PDE4B, whereas weak interactions with the M pocket of PDE4D suggest a lower inhibitory effect on PDE4D. These characteristics could be exploited to develop subfamily-specific inhibitors of PDE4. Future research should focus on designing inhibitor models based on these structural characteristics, conducting additional analyses through in silico docking studies, and confirming inhibitory effects through molecular biology experiments.

Furthermore, Table S2 lists many determined structures of members of the PDE4 family, most of which reveal structural information on them in complex with various chemical compounds. Although chemically bound PDE4 is not discussed here, other inhibitors such as rolipram and filaminast have also been reported to inhibit PDE4 activity. While the binding affinity or inhibitory potency of other compounds bound to PDE4 may be lower than that of roflumilast or cilomilast, the crystal structure of PDE4 complexed with other compounds still holds potential for use in developing a new generation of inhibitors. Therefore, future studies aimed at the development of PDE4 inhibitors should include a broader analysis of various chemicals capable of binding to PDE4.

## Figures and Tables

**Table 1:** Details of the crystal structures of PDE4 complexed with roflumilast and cilomilast.

Protein	Inhibitor	PDB Code	Resolution (Å)	Residues	Reference
PDE4B	Roflumilast	1XMU	2.30	324–700	[41]
	Cilomilast	1XLX	2.19	324–700	[41]
PDE4D	Roflumilast	1XOQ	1.83	388–715	[41]
	Roflumilast	3G4L	2.50	380–753	[65]
	Cilomilast	1XOM	1.55	388–715	[41]

## Data Availability

The model structure of the full length of canonical PDE4s generated by AlphaFold3 was deposited in ZENODO (https://zenodo.org/records/14405596).

## Supplementary Materials

The following supporting information can be downloaded at: www.mdpi.com/article/10.3390/molecules30030692/s1, Figure S1: Amino acid sequence alignment of PDE4A isoforms; Figure S2: Amino acid sequence alignment of PDE4B isoforms; Figure S3: Amino acid sequence alignment of PDE4C isoforms; Figure S4: Amino acid sequence alignment of PDE4D isoforms; Figure S5: Full-length model structure of canonical PDE4A, PDE4B, PDE4C, and PDE4D generated by AlphaFold3; Figure S6: Stereo view of the Q pocket of the catalytic domains of PDE4B and PDE4D; Figure S7: Stereo view of the M pocket of the catalytic domains of PDE4B and PDE4D; Table S1: Annotation and classification of PDE4 isoforms; Table S2: Experimentally determined PDE4 structures.
