# Supplementary material for: Molecular Properties of Phosphodiesterase 4 and Its Inhibition by Roflumilast and Cilomilast
Source: Molecules. 2025 Feb 4;30(3):692. doi: 10.3390/molecules30030692 (PMC11820465; doi:10.3390/molecules30030692)
Supplement: Supplementary file 1 [file molecules-30-00692-s001.zip › molecules-3408588-supplementary.pdf]

Supplementary Data

Molecular Properties of Phosphodiesterase 4 and Its Inhibition by Roflumilast and Cilomilast

Hyun Jeong Kwak [1,*] and Ki Hyun Nam [2,*]

[1] Department of Bio and Fermentation Convergence Technology, Kookmin University, Seoul 02707, Republic of Korea
[2] College of General Education, Kookmin University, Seoul 02707, Republic of Korea
* Correspondence: hjkwak@kookmin.ac.kr (H.J.K.); structure@kookmin.ac.kr (K.H.N.)

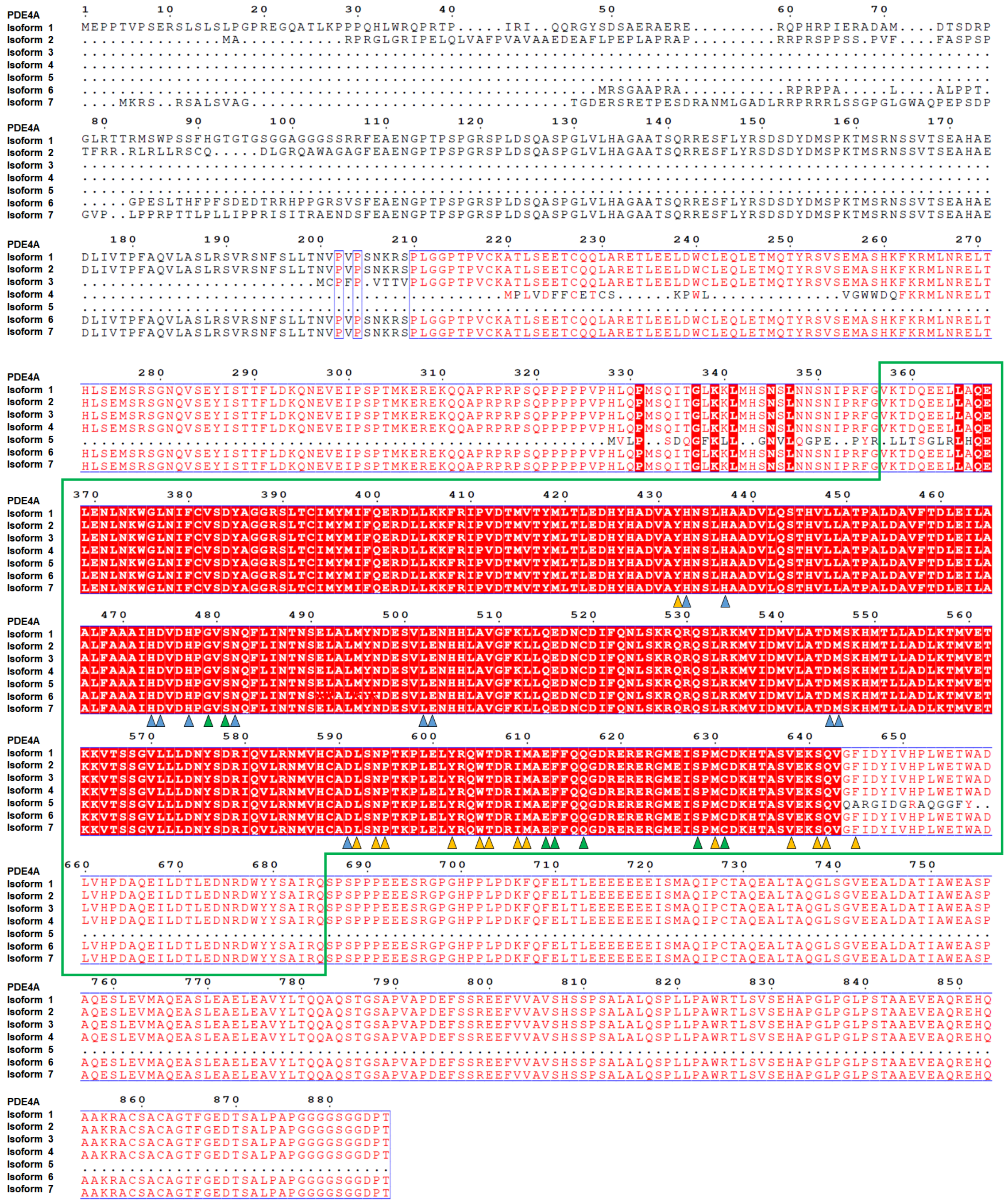

Supplementary Figure S1. Amino acid sequence alignment of PDE4A isoforms: isoform 1 (synonyms: PDE4A4/PDE4A4B/PDE46, UniProt code: P27815-1), isoform 2 (TM3/PDE4A11, P27815-2), isoform 3 (PDE4A7/PDE4A6, P27815-1), isoform 4 (PDE4A1/RD1, P27815-4), isoform 5 (PDE4A8A/2EL, P27815-5), isoform 6 (PDE4A10, P27815-6), and isoform 7 (PDE4A8, P27815-7). The residues involved in the metal binding pocket, Q switch and P clamp pocket, and solvent-filled side pocket are indicated by blue, yellow, and green triangles.

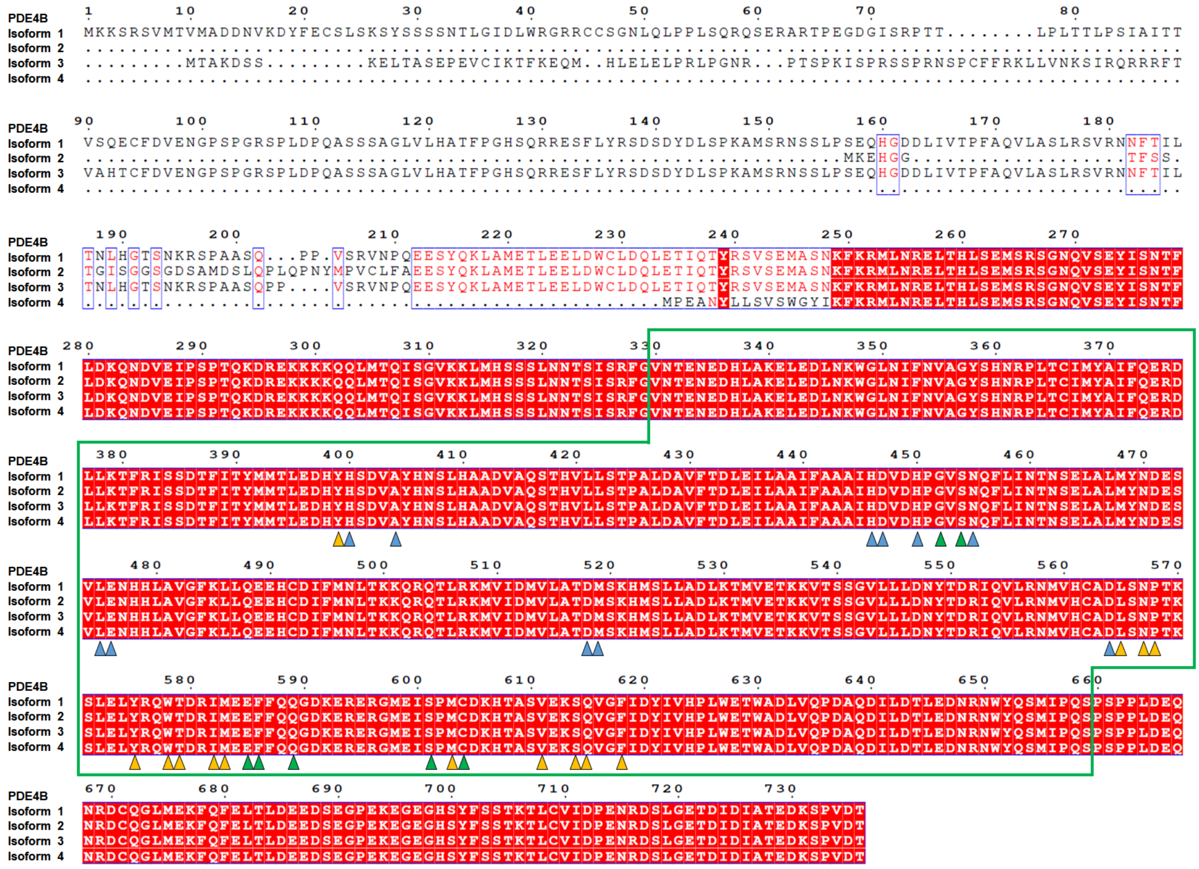

Supplementary Figure S2. Amino acid sequence alignment of the PDE4B isoforms: isoform 1 (synonym: PDE4B1, UniProt code: Q07343-1), isoform 2 (PDE4B2, Q07343-2), isoform 3 (PDE4B3, Q07343-3), and isoform 4 (PDE4B5, Q07343-4). The residues involved in the metal binding pocket, Q switch and P clamp pocket, and solvent-filled side pocket are indicated by blue, yellow, and green triangles.

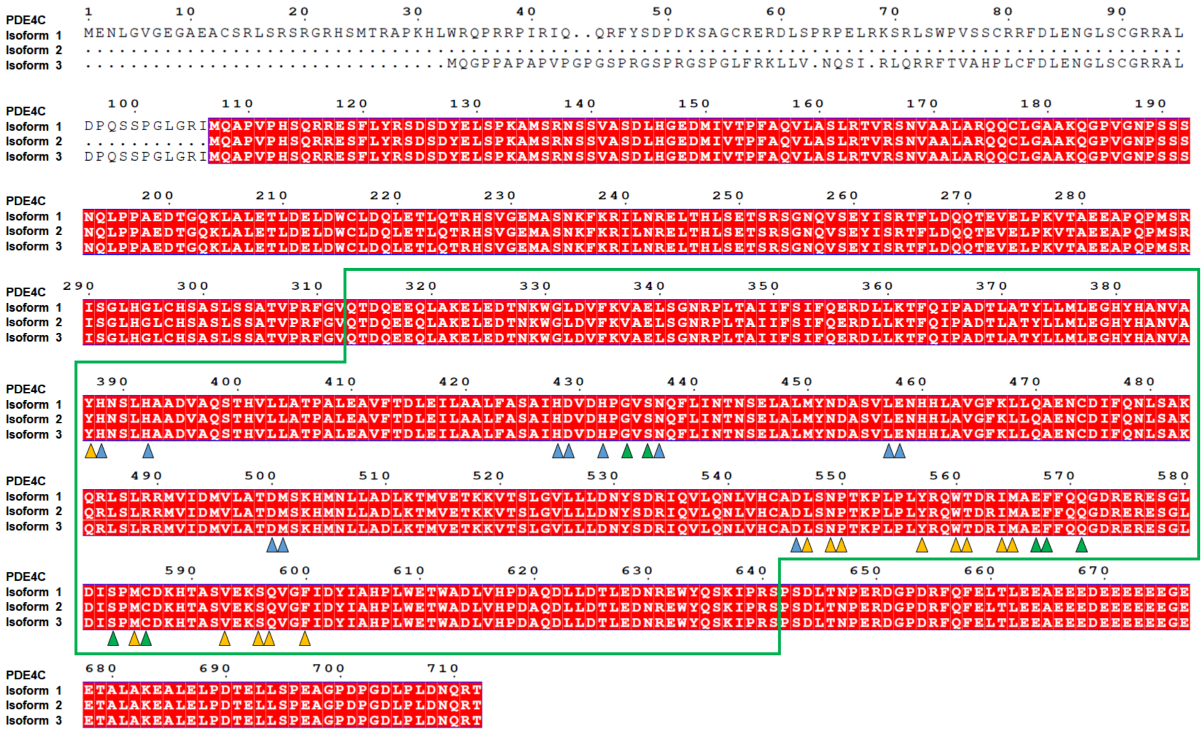

Supplementary Figure S3. Amino acid sequence alignment of PDE4C isoforms: isoform 1 (synonym: PDE4C1, UniProt code: Q08493-1), isoform 2 (PDE4C2, Q08493-2), isoform 3 (PDE4C3, Q08493-3), isoform 4 (PDE4C4, Q08493-4), isoform 5 (PDE4C5, Q08493-5), isoform 6 (PDE4C6, Q08493-6), and isoform 7 (PDE4C7, Q08493-7). The residues involved in the metal binding pocket, Q switch and P clamp pocket, and solvent-filled side pocket are indicated by blue, yellow, and green triangles.

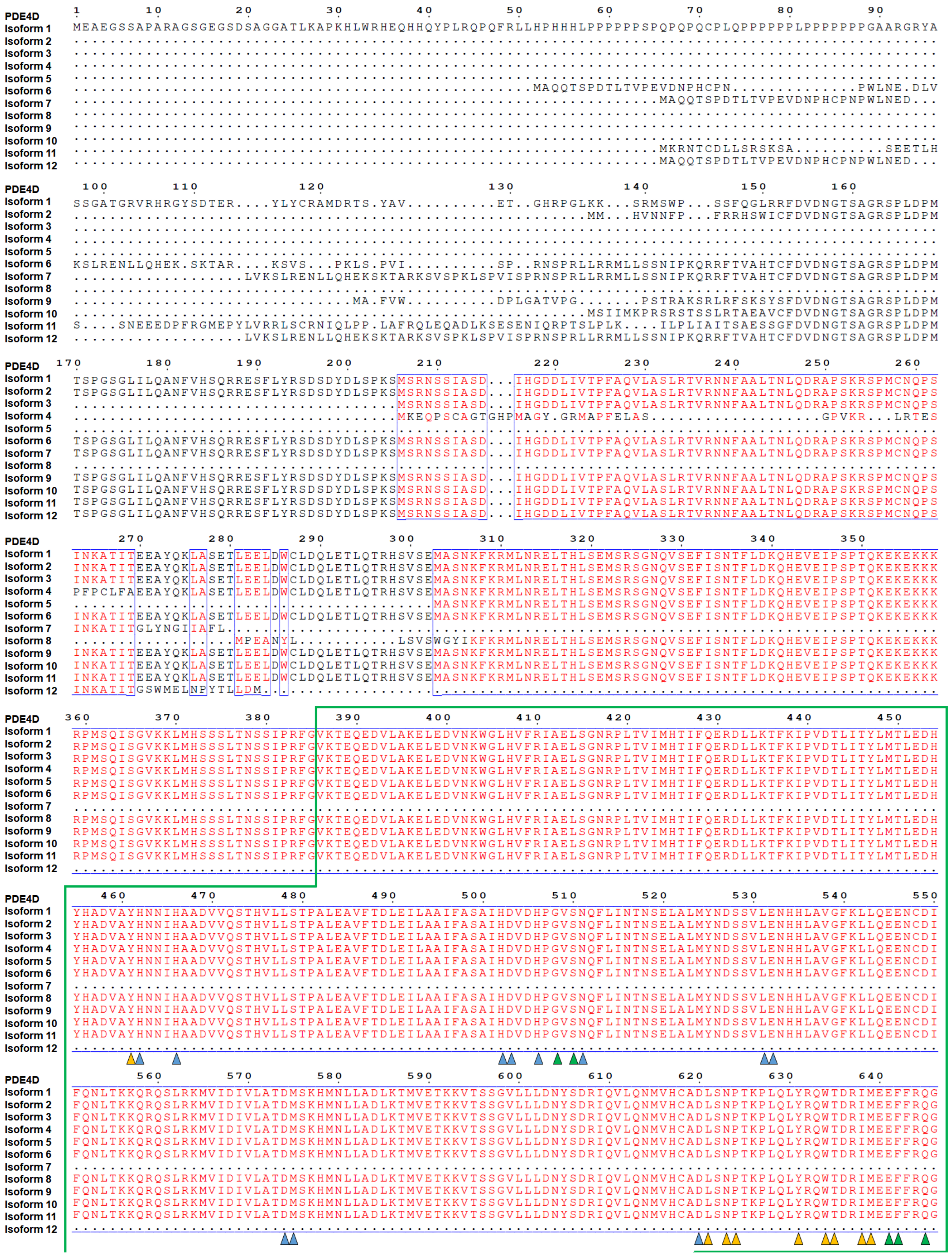

PDE4D
Isoform 1
Isoform 2
Isoform 3
Isoform 4
Isoform 5
Isoform 6
Isoform 7
Isoform 8
Isoform 9
Isoform 10
Isoform 11
Isoform 12

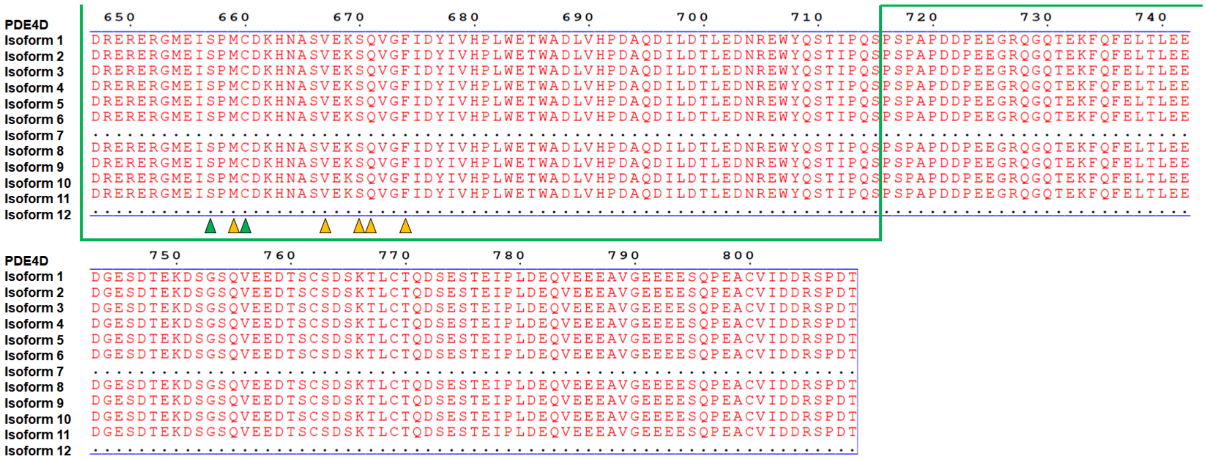

Supplementary Figure S4. Amino acid sequence alignment of PDE4D isoforms: isoform 1 (synonym: hPDE4D4, UniProt code: Q08499-1), isoform 2 (hPDE4D3, Q08499-2), isoform 3 (Q08499-3), isoform 4 (hPDE4D1, Q08499-4), isoform 5 (hPDE4D2, Q08499-5), isoform 6 (hPDE4D5, Q08499-6), isoform 7 (PDE4DN3, Q08499-7), isoform 8 (PDE4D6, Q08499-8), isoform 9 (PDE4D8, Q08499-9), isoform 10 (PDE4D9, Q08499-10), isoform 11 (PDE4D7, Q08499-11), and isoform 12 (Q08499-12). The residues involved in the metal binding pocket, Q switch and P clamp pocket, and solvent-filled side pocket are indicated by blue, yellow, and green triangles.

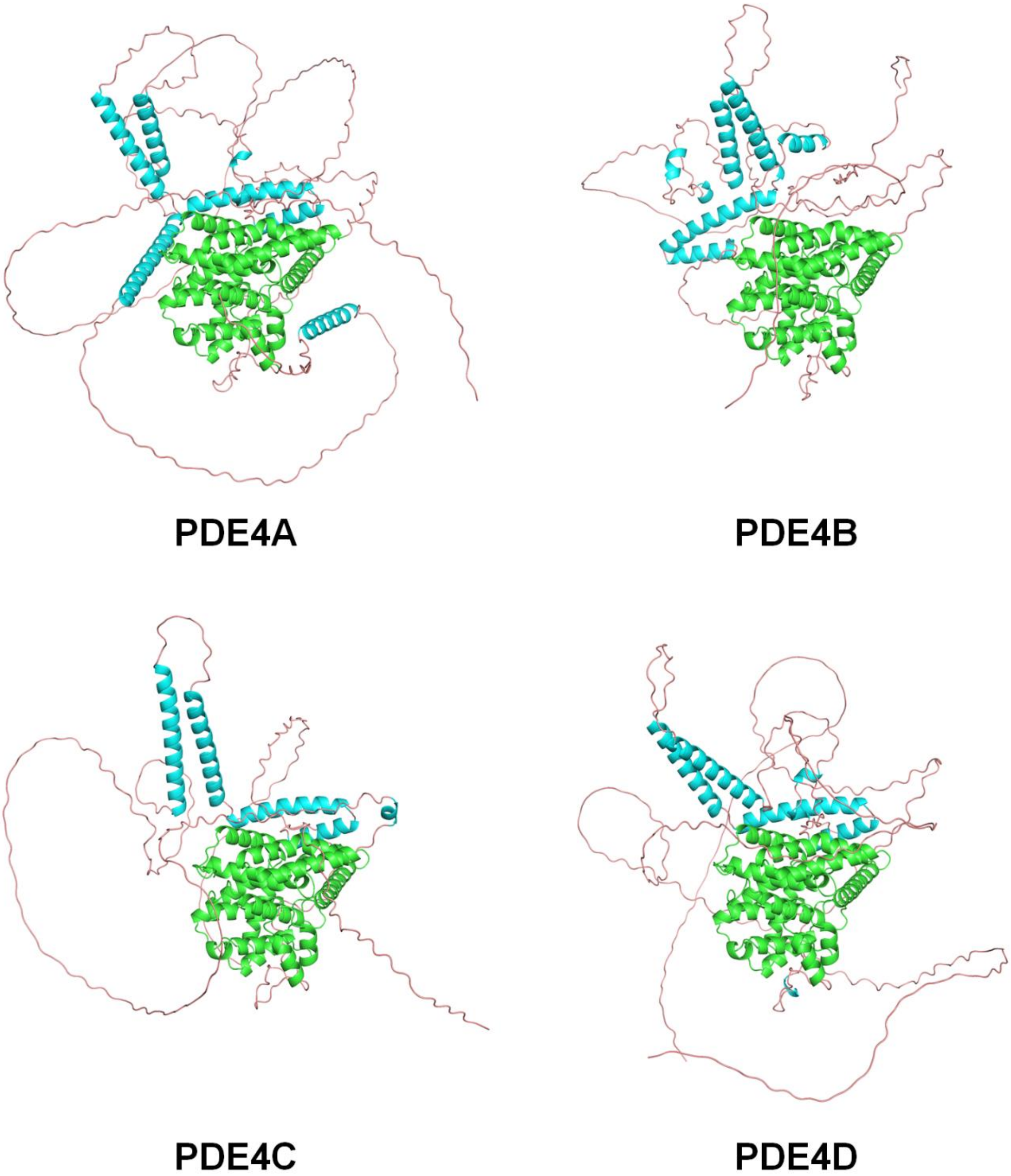

Supplementary Figure S5. Full-length model structure of canonical PDE4A, PDE4B, PDE4C, and PDE4D generated by AlphaFold3. The catalytic domain is indicated by green α-helices.

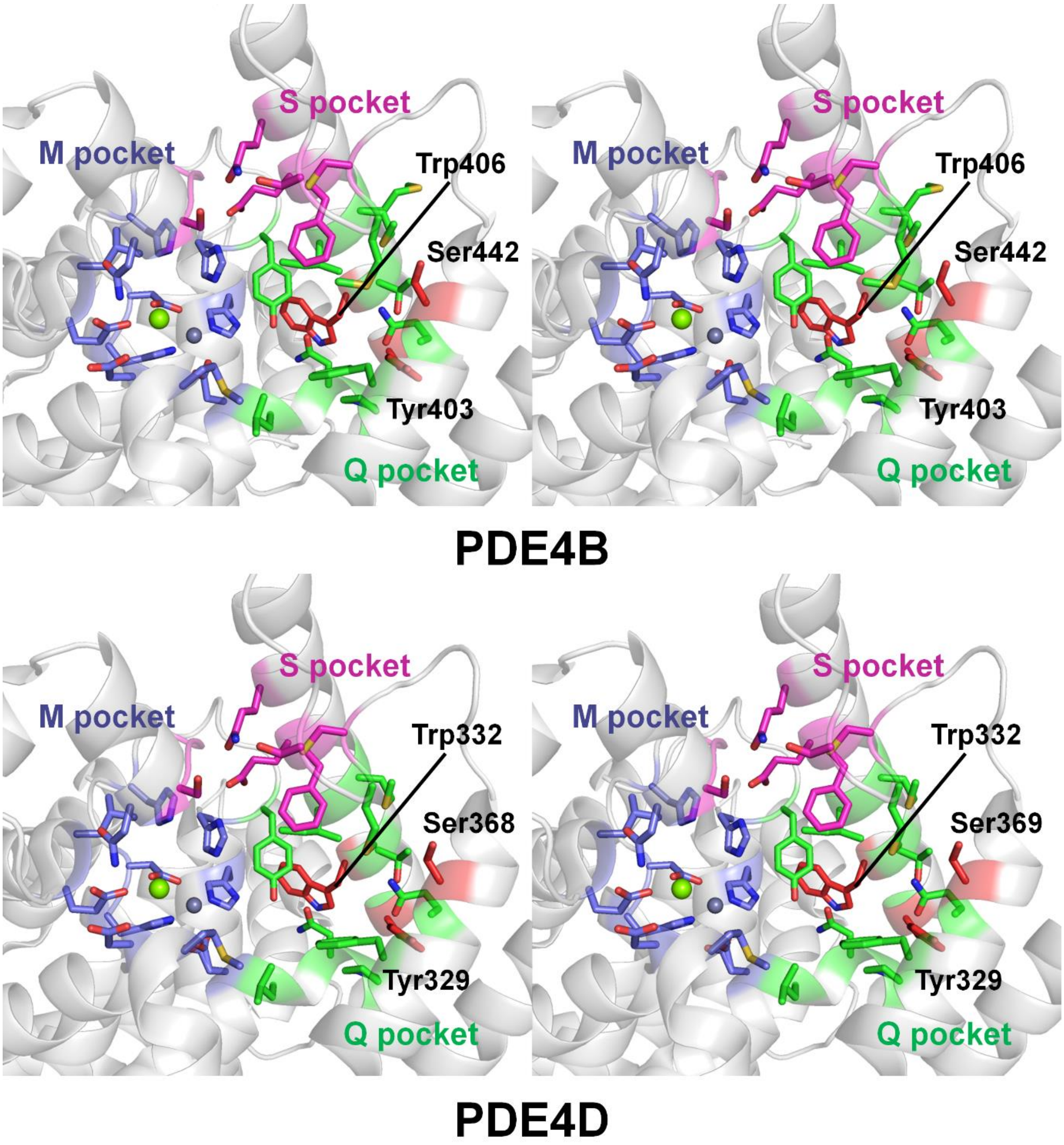

Supplementary Figure S6. Stereo view of the Q pocket (green) of the catalytic domains of PDE4B (PDB code: 1TB5) and PDE4D (PDB code: 1TB7). Tyr403, Trp406, and Ser442 (numbered in PDE4B) and Tyr329, Trp332, and Ser368 (numbered in PDE4D) are not involved in Q pocket formation.

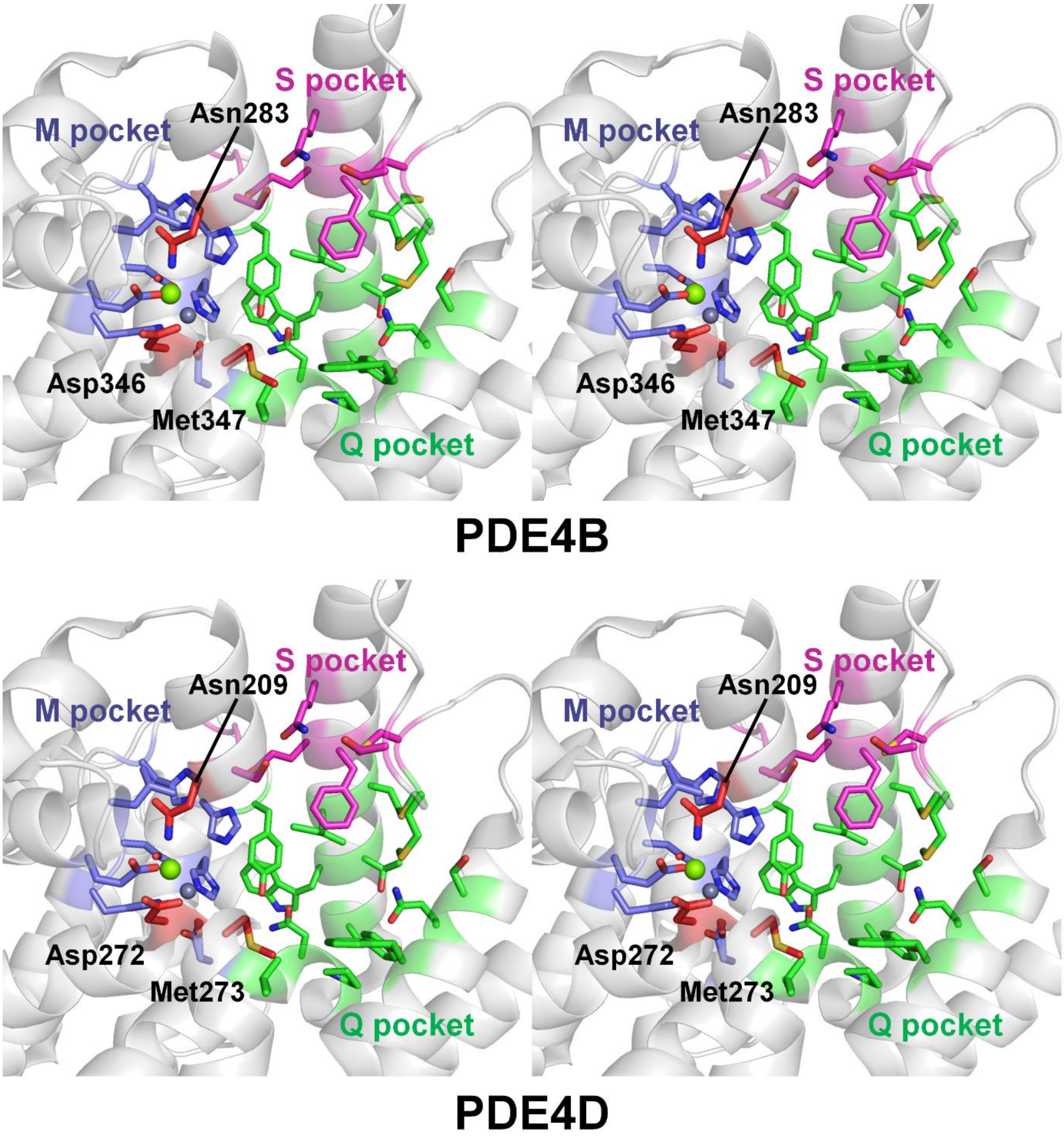

Supplementary Figure S7. Stereo view of the M pocket (blue) of the catalytic domains of PDE4B (PDB code: 1TB5) and PDE4D (PDB code: 1TB7). Asn283, Asp346, and Met347 (numbered in PDE4B) and Asn209, Asp272, and Met273 (numbered in PDE4D) are not involved in M pocket formation.

Table S1. Annotation and classification of PDE4 isoforms (UniProt)

Protein (UniProt Code)	Synonyms	Isoform (UniProt code)	Subcellular location *	Amino acids (kDa)
PDE4A (P27815)	PDE4A4, PDE4A4B, PDE46	Isoform 1 (P27815-1)	CYT, PNR	886 (98.143)
	TM3, PDE4A11	Isoform 2 (P27815-2)	CYT, PNR, CP, RM	860 (95.236)
	PDE4A7, PDE4A6	Isoform 3 (P27815-1)	CYT, CYTOS	686 (76.380)
	PDE4A1, RD1	Isoform 4 (P27815-4)	PMP	647 (72.222)
	PDE4A8A, 2EL	Isoform 5 (P27815-5)		323 (36,706)
	PDE4A10	Isoform 6 (P27815-6)	CYT, PNR	825 (91.323)
	PDE4A8	Isoform 7 (P27815-7)	CYT, CYTOS, PMP	864 (95.571)
PDE4B (Q07343)	PDE4B1	Isoform 1 (Q07343-1)		736 (83.343)
	PDE4B2	Isoform 2 (Q07343-2)		564 (64.352)
	PDE4B3	Isoform 3 (Q07343-3)		721 (82.096)
	PDE4B5	Isoform 4 (Q07343-4)	CYT, CM	503 (57.709)
PDE4C (Q08493)	PDE4C1	Isoform 1 (Q08493-1)		712 (79.902)
	PDE4C2	Isoform 2 (Q08493-2)		606 (67.791)
	PDE4C3	Isoform 3 (Q08493-3)		680 (75.614)
	PDE4C4	Isoform 4 (Q08493-4)		
	PDE4C5	Isoform 5 (Q08493-5)		
	PDE4C6	Isoform 6 (Q08493-6)		
	PDE4C7	Isoform 7 (Q08493-7)		
PDE4D (Q08499)	hPDE4D4	Isoform 1 (Q08499-1)		809 (91.115)
	hPDE4D3	Isoform 2 (Q08499-2)		673 (76.467)
		Isoform 3 (Q08499-3)		604 (68.607)
	hPDE4D1	Isoform 4 (Q08499-4)		584 (66.376)
	hPDE4D2	Isoform 5 (Q08499-5)		507 (57.792)
	hPDE4D5	Isoform 6 (Q08499-6)		745 (84.428)
	PDE4DN3	Isoform 7 (Q08499-7)		215 (23.839)
	PDE4D6	Isoform 8 (Q08499-8)		518 (59.113)
	PDE4D8	Isoform 9 (Q08499-9)		687 (77.705)
	PDE4D9	Isoform 10 (Q08499-10)		679 (76.816)
	PDE4D7	Isoform 11 (Q08499-11)		748 (84.662)
		Isoform 12 (Q08499-12)		219 (24.429)

* CYT: cytoplasm, CYTOS: cytosol, PMP: peripheral membrane protein, PNR: perinuclear region, CM: cell membrane, CP: cell projection, RM: ruffle membrane.

Table S2. Experimentally determined PDE4 structures.

PDE4 (UniProt ID)	PDB code (Ligand code)
PDE4A (P27815)	2QYK (NPV, Zn^{2+}, Mg^{2+}), 3HC8 (PD4, Zn^{2+}, Mg^{2+}), 3HDZ (PD6, Zn^{2+}, Mg^{2+}), 3I8V (0MO, Zn^{2+}, Mg^{2+}), 3TVX (PNX, Zn^{2+}, Mg^{2+}),
PDE4B (Q07343)	1F0J (Zn^{2+}, Mg^{2+}), 1RO6 (ROL, Zn^{2+}, Mn^{2+}), 1RO9 (8BR, Zn^{2+}), 1ROR (AMP, Zn^{2+}), 1TB5 (AMP, Zn^{2+}, Mn^{2+}), 1XLX (CIO, Zn^{2+}, Mn^{2+}), 1XLZ (FIL, Zn^{2+}, Mn^{2+}), 1XM4 (PIL, Zn^{2+}, Mn^{2+}), 1XM6 (5RM, Zn^{2+}, Mn^{2+}), 1XMU (ROF, Zn^{2+}, Mn^{2+}), 1XMY (ROL, Zn^{2+}, Mn^{2+}), 1XN0 (ROL, Zn^{2+}, Mn^{2+}), 1XOS (VIA, Zn^{2+}, Mn^{2+}), 1XOT (VDN, Zn^{2+}, Mg^{2+}), 1Y2H (6DE, Zn^{2+}, Mg^{2+}), 1Y2J (7DE, Zn^{2+}, Mg^{2+}), 2CHM (3P4, MES, Zn^{2+}, Mg^{2+}), 2QYL (NPV, Zn^{2+}, Mg^{2+}), 3D3P (20A, Zn^{2+}, Mg^{2+}), 3FRG (SK4, Zn^{2+}, Mg^{2+}), 3G45 (988, Zn^{2+}, Mg^{2+}), 3GWT (66, Zn^{2+}, Mg^{2+}), 3HMV (HBT, Zn^{2+}, Mg^{2+}), 3KKT (0CP, B3P, Zn^{2+}, Mg^{2+}), 3LY2 (Z72, SO4, Zn^{2+}, Mg^{2+}), 3O0J (3OJ, Zn^{2+}, Mg^{2+}), 3O56 (ZG1, Zn^{2+}, Mg^{2+}), 3O57 (ZG2, Zn^{2+}, Mg^{2+}), 3W5E (NVW, Zn^{2+}, Ca^{2+}), 3WD9 (QPC, Zn^{2+}, Ca^{2+}), 4KP6 (1S1, Zn^{2+}, Mg^{2+}), 4MYQ (19T, Na+, Zn^{2+}, Mg^{2+}), 4NW7 (2O5, Zn^{2+}, Mg^{2+}, Na^{+}), 4WZI (Zn^{2+}, Mg^{2+}), 4X0F (ROL Zn^{2+}, Mg^{2+}), 5K6J (6QQ, Zn^{2+}, Mg^{2+}), 5LAQ (6M5,ACT, Zn^{2+}, Mg^{2+}), 5OHJ (9VE Zn^{2+}, Mg^{2+}), 6BOJ (E31, Zn^{2+}, Mg^{2+}, Cl^{-}), 8OEG (VL9, MPD Zn^{2+}, Mg^{2+})
PDE4C (Q08493)	2QYM (Zn^{2+}, Mg^{2+})
PDE4D (Q08499)	1E9K, 1MKD (ZAR, Zn^{2+}, Mg^{2+}), 1OYN (ROL, Zn^{2+}), 1PTW (AMP, Zn^{2+}), 1Q9M (ROL, Zn^{2+}), 1TB7 (AMP, B3P, Zn^{2+}, Mg^{2+}), 1TBB (ROL, Zn^{2+}, Mg^{2+}), 1XOM (CIO, Zn^{2+}, Mg^{2+}), 1XON (B3P, PIL, Zn^{2+}, Mg^{2+}), 1XOQ (ROF, Zn^{2+}, Mg^{2+}), 1XOR (ZAR, Zn^{2+}, Mg^{2+}), 1Y2B (DEE, Zn^{2+}, Mg^{2+}), 1Y2C (3DE, Zn^{2+}, Mg^{2+}), 1Y2D (4DE, B3P, Zn^{2+}, Mg^{2+}), 1Y2E (5DE, Zn^{2+}, Mg^{2+}), 1Y2K (7DE, Zn^{2+}, Mg^{2+}), 1ZKN (IBM, Zn^{2+}, Mg^{2+}), 2FM0 (M98, Zn^{2+}, Mg^{2+}), 2FM5 (M99, Zn^{2+}, Mg^{2+}), 2PW3 (CMP, Zn^{2+}), 2QYN (NPV, Zn^{2+}, Mg^{2+}), 3G4G (D71, Zn^{2+}, Mg^{2+}, Ca^{2+}), 3G4I (D71, EOH, Zn^{2+}, Mg^{2+}), 3G4K (ROL, Zn^{2+}, Mg^{2+}), 3G4L (ROF, Zn^{2+}, Mg^{2+}), 3G58 (988, Zn^{2+}, Mg^{2+}), 3IAD (15X, Zn^{2+}, Mg^{2+}), 3IAK (EV1, Zn^{2+}, Mg^{2+}), 3K4S (0MO, Zn^{2+}, Mg^{2+}), 3SL3 (DMS, EDO, EPE, PEG, PO4, Zn^{2+}), 3SL4 (JN4, PO4, Zn^{2+}), 3SL5 (J25, Zn^{2+}), 3SL6 (JN8, Zn^{2+}), 3SL8 (JN7, PO4, Zn^{2+}), 3V9B (IHM, Zn^{2+}), 4OGB (2SR, Zn^{2+}), 4W1O (3GJ, Zn^{2+}), 4WCU (3KQ, Zn^{2+}, Mg^{2+}), 5K1I (6PT, Zn^{2+}, Mg^{2+}), 5K32 (6Q2, Zn^{2+}, Mg^{2+}), 5LBO (6M5, DTT,, Zn^{2+}, Mg^{2+}), 5TKB (7DJ, EOH, Zn^{2+}, Mg^{2+}), 5WH5 (R91, Zn^{2+}, Mg^{2+}), 5WH6 (AKJ, Zn^{2+}, Mg^{2+}), 5WQA (J20, Zn^{2+}, Mg^{2+}), 6AKR (A0O, Zn^{2+}), 6BOJ (E31, Cl^{-}, MPD, Zn^{2+}, Mg^{2+}), 6F6U (CV8, GOL, Zn^{2+}, Mg^{2+}), 6F8R (CZK, Zn^{2+}, Mg^{2+}), 6F8T (CZT, Zn^{2+}, Mg^{2+}), 6F8U (CZQ, Zn^{2+}, Mg^{2+}), 6F8V (D0B, Zn^{2+}, Mg^{2+}), 6F8W (D0E, Zn^{2+}, Mg^{2+}), 6F8X (D08, Zn^{2+}, Mg^{2+}), 6FDC (DD5, D5N, Zn^{2+}, Mg^{2+}), 6FDI (D5T, Zn^{2+}, Mg^{2+}), 6FE7 (D62, Zn^{2+}, Mg^{2+}), 6FEB (D5Z, Zn^{2+}, Mg^{2+}), 6FET (D68, Zn^{2+}, Mg^{2+}), 6FT0 (E6E, Zn^{2+}, Mg^{2+}), 6FTA (E6N, Zn^{2+}, Mg^{2+}), 6FTW (E6Z, Zn^{2+}, Mg^{2+}), 6FW3 (E8H, Zn^{2+}, Mg^{2+}), 6HWO (FFZ, Zn^{2+}, Mg^{2+}), 6IAG (E3Q, Zn^{2+}, Mg^{2+}), 6IBF (4I7, Zn^{2+}, Mg^{2+}), 6IM6 (AH3, Zn^{2+}, Mg^{2+}), 6IMB (AH9, Zn^{2+}, Mg^{2+}), 6IMD (AH9, Zn^{2+}, Mg^{2+}), 6IMI (AH6, Zn^{2+}, Mg^{2+}), 6IMO (AJL, Zn^{2+}, Mg^{2+}), 6IMR (AJX, Zn^{2+}, Mg^{2+}), 6IMT (AK0, Zn^{2+}, Mg^{2+}), 6IND (AKO, Zn^{2+}, Mg^{2+}), 6INK (AKU, Zn^{2+}, Mg^{2+}), 6INM (AKU, Zn^{2+}, Mg^{2+}), 6KJZ (MKU, Zn^{2+}, Mg^{2+}), 6KK0 (M36, Zn^{2+}, Mg^{2+}), 6LRM (EQC, Zn^{2+}, Mg^{2+}), 6NJH (KRD, Zn^{2+}, Mg^{2+}), 6NJI (KR4, Zn^{2+}, Mg^{2+}), 6NJJ (KR7, BTB, Zn^{2+}, Mg^{2+}), 6RCW (DTT, JX2, Zn^{2+}, Mg^{2+}), 6ZBA (QDT, Zn^{2+}, Mg^{2+}), 7A8Q (R4H, Zn^{2+}, Mg^{2+}), 7A9V (R5Z, Zn^{2+}, Mg^{2+}), 7AAG (QWT, Zn^{2+}, Mg^{2+}, ILE, PRO), 7AB9 (QWZ, Zn^{2+}, Mg^{2+}), 7ABD (RLW, Zn^{2+}, Mg^{2+}), 7ABE (J2E, Zn^{2+}, Mg^{2+}), 7ABJ (863, Zn^{2+}, Mg^{2+}), 7AY6 (S8Q, Zn^{2+}, Mg^{2+}), 7B9H (T3K, Zn^{2+}, Mg^{2+}), 7CBJ (FTX, Zn^{2+}, Mg^{2+}), 7CBQ (A9L, Zn^{2+}, Mg^{2+}), 7F2K (0X8, Zn^{2+}, Mg^{2+}), 7F2L (1AS, Zn^{2+}, Mg^{2+}), 7F2M (1GF, Zn^{2+}, Mg^{2+}), 7W4X (8G7, Zn^{2+}, Mg^{2+}), 7W4Y (8GO, Zn^{2+}, Mg^{2+}), 7XAA (AQL, Zn^{2+}, Mg^{2+}), 7XAB (AWI, Zn^{2+}, Mg^{2+}), 7XBB (B6V, Zn^{2+}, Mg^{2+}), 7YQF (JN0, Zn^{2+}, Mg^{2+}), 7YSX (JU3, Zn^{2+}, Mg^{2+}), 8K4C (VIC, Zn^{2+}, Mg^{2+}), 8K4H (R75, Zn^{2+}, Mg^{2+}), 8W4Q (3NG, Zn^{2+}, Mg^{2+}), 8W4R (AJR, Zn^{2+}, Mg^{2+}), 8YLC (A1D6Q, Zn^{2+}, Mg^{2+}),

* The PDE4-bound ligands delivered from the crystallization solution were removed from the PDB list.
